# Synthetic Polymer Aerogels in Particulate Form

**DOI:** 10.3390/ma12091543

**Published:** 2019-05-10

**Authors:** Patrina Paraskevopoulou, Despoina Chriti, Grigorios Raptopoulos, George C. Anyfantis

**Affiliations:** Laboratory of Inorganic Chemistry, Department of Chemistry, National and Kapodistrian University of Athens, Panepistimiopolis Zografou, 15771 Athens, Greece; chritides@chem.uoa.gr (D.C.); grigorisrap@chem.uoa.gr (G.R.); gc.anyfantis@gmail.com (G.C.A.)

**Keywords:** aerogels, polyurea, polyimide, phenolic resin, polybenzoxazine, resorcinol-formaldehyde, particles, beads

## Abstract

Aerogels have been defined as solid colloidal or polymeric networks of nanoparticles that are expanded throughout their entire volume by a gas. They have high surface areas, low thermal conductivities, low dielectric constants, and high acoustic attenuation, all of which are very attractive properties for applications that range from thermal and acoustic insulation to dielectrics to drug delivery. However, one of the most important impediments to that potential has been that most efforts have been concentrated on monolithic aerogels, which are prone to defects and their production requires long and costly processing. An alternative approach is to consider manufacturing aerogels in particulate form. Recognizing that need, the European Commission funded “NanoHybrids”, a 3.5 years project under the Horizon 2020 framework with 12 industrial and academic partners aiming at aerogel particles from bio- and synthetic polymers. Biopolymer aerogels in particulate form have been reviewed recently. This mini-review focuses on the emerging field of particulate aerogels from synthetic polymers. That category includes mostly polyurea aerogels, but also some isolated cases of polyimide and phenolic resin aerogels. Particulate aerogels covered include powders, micro granules and spherical millimeter-size beads. For the benefit of the reader, in addition to the literature, some new results from our laboratory concerning polyurea particle aerogels are also included.

## 1. Introduction

Aerogels have been defined as solid colloidal or polymeric networks of nanoparticles expanded throughout their entire volume by a gas [1,2]. With silica aerogels as a well-known example, aerogels have been called “frozen smoke”, and in practice they are highly open porous ultra-lightweight materials [3,4,5], consisting of up to more than 90% v/v of empty space. They are prepared by removing pore-filling solvents from wet-gels without substantial volume reduction or network compaction. Typically, that is carried out by turning the pore-filling solvent into a supercritical fluid that is released slowly like a gas. That process allows aerogels to retain the structural shape of their wet-gel precursors [5].

Silica aerogels were first prepared by S. S. Kistler in the 1930s [6,7]. Besides silica, he successfully prepared other metal oxide aerogels along with some organic aerogels [8,9]. Kistler’s first silica aerogels were commercialized through Monsanto Chemical Company [10]. However, time-consuming gelation and solvent exchanges were the main drawbacks of Kistler’s method. A new method for aerogels synthesis using alkoxides as precursors was published by J. B. Peri in 1966 [11]. Research efforts have subsequently extended this class of materials to non-silica inorganic oxides, natural and synthetic organic polymers, carbons, metals and ceramic materials [12].

As with any porous material, the bulk physical properties are influenced by the size and shape of the pores. Most aerogels are mesoporous materials with pore sizes in the 2–50 nm range. The solid network has a complex hierarchical structure coming from aggregation of smaller primary particles to fractal porous secondary particles, eventually agglomerating to a structure resembling a “pearl-necklace”. The finely structured porous skeletal framework together with the small size pores provides aerogels with high surface areas, low thermal conductivities, low dielectric constants, and high acoustic attenuation [4,13,14,15]. Those properties make aerogels promising materials for a wide variety of areas, including, but not limited to, energy storage [16,17,18,19], thermal [18,20,21,22,23,24,25] and acoustic insulation [20,21], dielectrics [20], gas and humidity adsorption [20,24,26], for space applications [20], gas sensors [26], environmental remediation [27,28], catalysis [4,17,24,26,29,30,31], biomedicine [19,25,32], and the food industry [24,32]. However, monolithic aerogels are prone to cracks and defects [33] and their production requires long processing times, which may hinder their potential. One way toward efficient preparation and processing is to consider manufacturing aerogels in particulate rather than in monolithic form [34]. In response to that need, the European Commission is funding “NanoHybrids” [35], a 3.5 year program under the Horizon 2020 framework with 12 industrial and academic partners aiming at the development of technology for aerogel particles production from bio- and synthetic polymers. The production of biopolymer aerogels in particulate form [36,37,38] has been reported in recent publications. Synthetic polymer aerogels in powder, micro granule and spherical millimeter-size bead form are reviewed here.

Silica aerogels, arguably the archetypical example of aerogels, have been synthesized as monoliths (relatively difficult to manufacture in cm-size and above), composite blankets (the main product of the aerogel thermal insulation industry), and as powders and granules (also commercially available) [39]. For certain applications, particulate aerogels are preferable over monoliths. For example, silica aerogel microspheres (120 ± 30 μm in diameter; mean pore size: 14 nm) perform better than silica aerogel monoliths toward filtration of air to remove latex spheres (20–2000 nm) [40], and silica aerogel granules (150–250 μm and 50–500 μm) outperform high efficiency particulate air (HEPA) filters in solid and oily aerosol collection [41]. Silica aerogel granules (0.01–4 mm) have also shown high thermal insulation (thermal conductivity as low as 0.019 W m^−1^ K^−1^) as well as acoustic insulation (transmission loss as high as 15 dB at 1700 Hz) properties, both improving with decreasing size and increasing packing density of the granules [42]. Hollow and granular silica aerogel microspheres have been prepared from rice husk ash and have found applications in drug delivery [43].

Silica aerogel microparticles (155 μm to 1.7 mm) have been synthesized from tetramethylorthosilicate via emulsion gelation and CO_2_ supercritical extraction [44]. Silica aerogel granules have been prepared from polyethoxysiloxanes in a one-pot process requiring minimal amounts of solvent and processing time [45]. Uniform mm-sized silica aerogel beads have been synthesized from sodium silicate and tetraethoxysilane (hydrophobic beads), or from trimethylchlorosilane (hydrophilic beads) [46], or from tetraethoxysilane by extrusion of the silica sol into an oil phase through nozzles [47].

Carbon aerogel beads have been prepared by pyrolysis of resorcinol-formaldehyde [48,49,50,51,52,53] or polybenzoxazine [54] beads. This kind of aerogels are chemically inert, have high electric conductivity and adjustable internal characteristics; thus, they can find applications in electrodes or in supercapacitors [49,51,52], with specific capacitance as high as 215 F g^–1^ [49]. They have also been applied as adsorbents for phenol, with adsorption capacity of 29.3 mg g^–1^ [53].

In that regard, although there is a vast literature for monolithic aerogels derived from synthetic polymers, including aerogels based on phenolic resins (e.g., resorcinol-formaldehyde [19,55,56,57,58,59,60,61], polybenzoxazines [62,63,64,65,66], polyureas [67,68,69], polyimides [70,71,72,73,74], polyamides (Kevlar^TM^-like) [74,75,76], polyurethanes [77,78,79,80,81,82,83], as well as aerogels derived via Ring Opening Metathesis Polymerization (ROMP), including polynorbornene and polydicyclopentadiene [81,84,85,86,87], only a few examples of synthetic polymer aerogel particles are known, and they have become the subject of this mini-review. Those polymeric materials include mostly polyurea aerogels, and a few examples of polyimide and phenolic resin aerogels. Phenolic resin aerogel beads were synthesized as precursors of carbon aerogels in bead form, and characterization was mainly focused on the carbons.

## 2. Polyurea (PUA) Aerogels

**Scheme 1:**
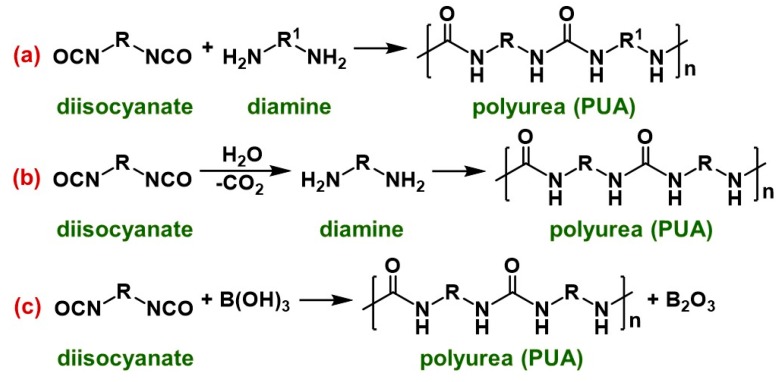
Synthesis of PUA aerogels from isocyanates and amines (**a**), isocyanates and water (**b**), or isocyanates and boric acid (**c**).

Polyureas (PUA) are a class of polymers that can be defined as the product of the reaction between an isocyanate and an amine, as shown in Scheme 1a. PUA can be a good elastomer, with good mechanical properties, chemical stability, thermal shock abrasion resistance, flexibility and water repellence. Those properties depend on the chemical identity and structure of isocyanates and amines, hydrogen bonding and the polymerization conditions. The nucleophilic addition of the amine to the isocyanate is fast and catalyst-free (aromatic isocyanates are more reactive than aliphatic ones because of the electron-withdrawing properties of the aromatic groups), which are advantages for the production of aerogels using sol-gel processing.

**Scheme 2:**
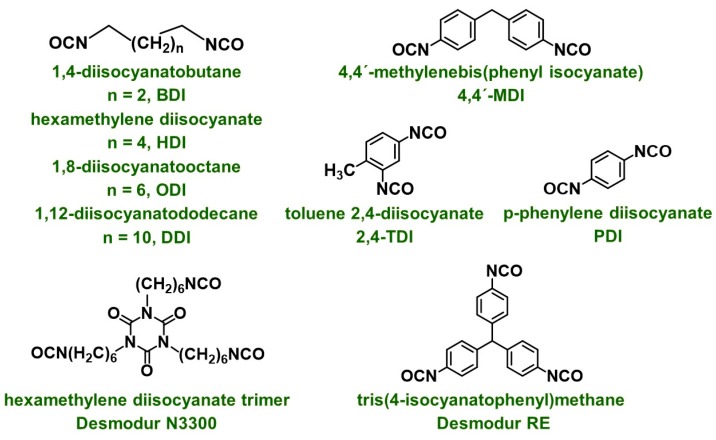
Structures of the di- and triisocyanates used for the synthesis of the materials discussed in this review.

In a more economical approach, PUA can also be formed from the reaction of isocyanates with water, as shown in Scheme 1b [67]. Isocyanates and water form unstable carbamic acids, which decompose by eliminating carbon dioxide and yield amines. Those amines react with the remaining isocyanates yielding ureas. Although this route to ureas needs a catalyst (triethylamine), it is advantageous because it bypasses the use of multifunctional amines, which can be expensive. Aerogels prepared from the reaction of triisocyanate Desmodur N3300 (Scheme 2) with water showed that their nanomorphology depended on the concentration of the isocyanate in the sol and was nearly independent of the concentrations of water and catalyst, which mainly affected the gelation time and the density of the materials. By replacing the triisocyanate Desmodur N3300 with the more rigid aromatic Desmodur RE (Scheme 2), the skeletal framework seemed more particulate than fibrous, even at lower densities. Additionally, variations on the nanomorphology of the porous samples can be achieved with different gelation solvents [68].

Another approach is the synthesis of PUA aerogels from the reaction of Desmodur RE with anhydrous H_3_BO_3_ (Scheme 1c) [69]. The by-product, B_2_O_3_, is soluble and could be easily washed off the porous network, leaving behind pure PUA. The same reaction proceeded with a number of mineral acids, such as H_3_PO_4_, H_3_PO_3_, H_2_SeO_3_, H_6_TeO_6_, H_5_IO_6_ and H_3_AuO_3_, but the corresponding oxides are insoluble and remained within the polymer. PUA aerogels obtained with this method were chemically identical, except in minute details at the nanoscopic level, to PUA aerogels obtained via reaction of Desmodur RE with water. That reaction pathway is distinctly different from the conventional path followed by isocyanates with carboxylic acids to amides, and has not been explored for particulate PUA aerogels yet. However, that approach is highly promising for metal- and metal-salt doped PUA aerogel beads and carbons and is currently under investigation.

### 2.1. PUA Aerogel Powders and Granules

**Scheme 3:**
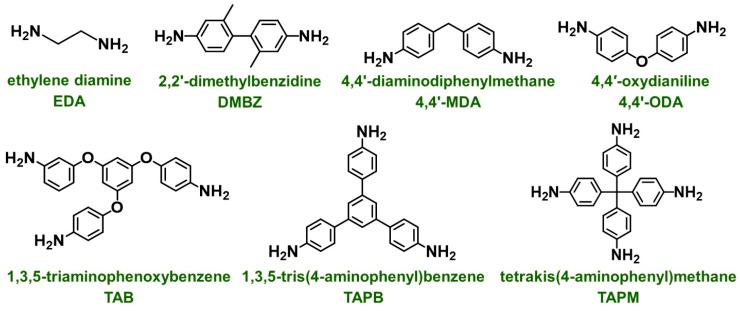
Structures of the di- and tetraamines used for the synthesis of the materials discussed in this review.

**Figure 1:**
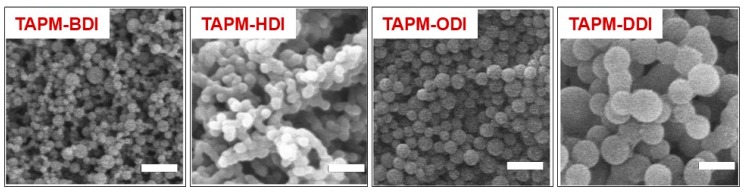
SEM images of PUA powders precipitated into acetone from the sols of the monomer pairs, as indicated, at 50% reaction conversion. Initial solution concentration: 0.04 g mL^–1^. Scale bar: 400 nm. Adapted from Ref. [89] with permission from The Royal Society of Chemistry.

Several PUA powders with aerogel-like internal structure have been synthesized via reaction of tetrakis(4-aminophenyl)methane (TAPM; Scheme 3) with a variety of alkyl diisocyanates (1,4-diisocyanatobutane (BDI), 1,6-diisocyanatohexane (HDI), toluene 2,4-diisocyanate (2,4-TDI), 1,8-diisocyanatooctane (ODI), 1,12-diisocyanatododecane (DDI), 4,4′-methylenebis(phenyl isocyanate) (MDI), *p*-phenylene diisocyanate (PDI); Scheme 2) in DMF at room temperature (r.t.), followed by precipitation induced by addition of a large volume of a non-solvent (acetone) just before gelation [88,89]. The precipitate was washed 3 times with acetone and was dried at 150 °C under vacuum. The product consisted of spherical particles, the size of which was larger (on the order of hundreds of nm—see Figure 1) than the gel network nanoparticles (about 9–30 nm, measured with dynamic light scattering) and depended on the chemical identity of the diisocyanate. The largest particles were obtained with the isocyanate with the largest aliphatic chain (DDI). Pore sizes were in the range of 5–9 Å, with higher percentage in the range of 5–6 Å for BDI and HDI derived samples. BET surface areas measured with N_2_ sorption (19–68 m^2^ g^–1^) were much lower than surface areas measured with CO_2_ sorption (170–240 m^2^ g^–1^). On geometric grounds, and assuming a density of 1 g cm^–3^, it was calculated that BET surface areas corresponded to the external surface area of the particles shown in SEM. Therefore, it was concluded that micropores could be sampled only by CO_2_, and not by N_2_. Based on those differential gas sorption characteristics, it was proposed that such PUA powders are suitable for application in gas separations (e.g., CO_2_, N_2_ and CH_4_). The best separations were achieved with TAPM-BDI derived PUA powder, which had a CO_2_/N_2_ selectivity of 65.5 mol mol^–1^ and a CO_2_/CH_4_ selectivity of 8.3 mol mol^–1^.

**Figure 2:**
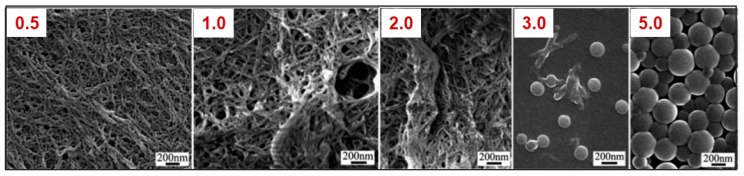
SEM images of the polymers prepared from the reaction of 2,4-TDI and 4,4′-ODA in acetone at 30 °C for 4 h under reciprocating shaking (150 oscillations per min), at five different monomer concentrations (% w/w), as indicated. Adapted from Ref. [90] with permission from The Royal Society of Chemistry.

**Figure 3:**
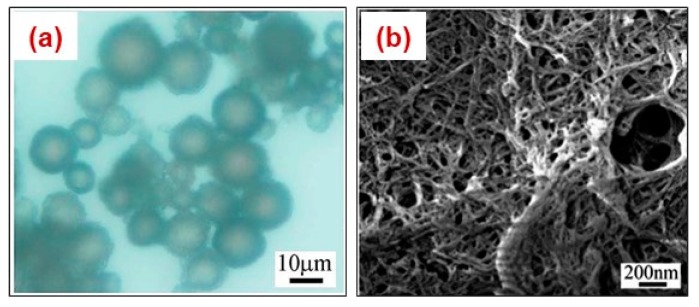
SEM images of PUA particles prepared at 30 °C in acetonitrile (**a**), and in acetone (**b**) under reciprocating shaking (150 oscillations per minute). Monomer concentration: 1.0% w/w [90]. Adapted from Ref. [90] with permission from The Royal Society of Chemistry.

**Scheme 4:**
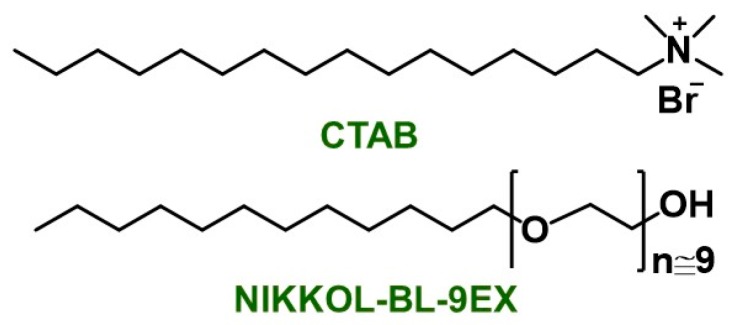
Structures of surfactants CTAB and NIKKOL BL-9EX.

PUA powders with aerogel-like internal structure have been also produced by varying the conditions of mechanical agitation at 30 °C of sols from 2,4-TDI (Scheme 2) and 4,4′-oxydianiline (4,4′-ODA; Scheme 3) in acetone, and in selected cases also in acetonitrile [90]. The reaction was run always under conditions of precipitation polymerization. The typical total monomer concentration (2,4-TDI+4,4′-ODA) in the sol was 1% w/w. Resulting powders were compared among themselves, *viz.* with no reference to monolithic aerogels. Precipitates were collected by centrifugation, washed with acetone and dried at 60 °C to powdery products. No application of vacuum during drying was reported. As-prepared PUA powders were thermally stable (degradation started at 260 °C), and insoluble in several solvent systems at 70 °C under reciprocating shaking for 6 h. Those solvent systems included acetic acid, toluene, acrylonitrile, *m*-cresol, THF, a mixture of HCl_(aq)_/acetone (1:1 v/v) and aqueous alkali (1.0 mol L^–1^). Fibrous morphologies with fiber diameters ≤100 nm were reported under conditions without stirring, stirring at low stirring rates (100 rpm), or with reciprocating stirring. Higher stirring rates (600 rpm) produced granular polymers. Nanomorphology, however, was a complicated function of several parameters: For example, with no stirring at all, the network nanomorphology changed from fibrous to particulate (granular aggregates) by lowering the reaction temperature from 30 to 0 °C. Similarly, using reciprocated stirring, nanomorphology changed from fibrous to particulate by increasing the total monomer concentration (2,4-TDI+4,4′-ODA) from 1 to 5% w/w (Figure 2). Nanofibers were obtained with monomer concentrations lower than 2% w/w (Figure 2). Running the reaction in acetonitrile (all other parameters being the same, i.e., reciprocating stirring, [2,4-TDI+4,4′-ODA] = 1% w/w, 30 °C) yielded large (5–20 μm in diameter) spherical particles rather than fibers (Figure 3). Switching from fibers in acetone [67] to large micron-size particles in acetonitrile [68] has also been reported with monolithic PUA aerogels synthesized from Desmodur N3300, an aliphatic triisocyanate, and water. Overall, all morphological changes observed in PUA aerogels as a function of solvent and monomer concentration have been attributed recently to the kinetics and the mechanism of the gelation process, that is phase separation of solid colloidal particles versus liquid oligomers [91].

**Figure 4:**
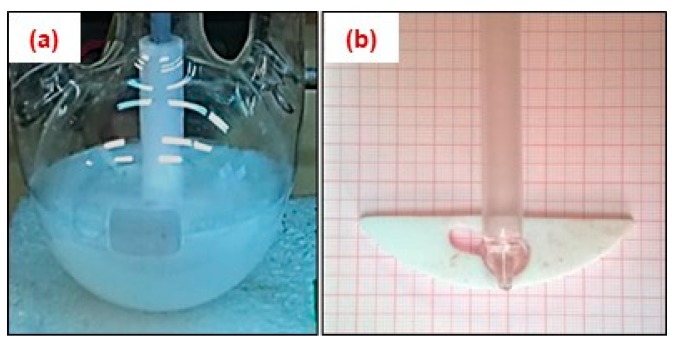
(**a**) Experimental setup for the synthesis of PUA aerogel powders with disruption of gelation (vigorous agitation, 250–300 rpm; suspension and emulsion polymerization, 400 rpm). (**b**) The half-moon shaped blade used for agitation.

PUA aerogel powders have been fabricated from the reaction of Desmodur N3300 (Scheme 2) with water in organic solvents (acetone or propylene carbonate). Three methods have been employed: vigorous agitation of the sol, suspension, and emulsion gelation, using the experimental setup shown in Figure 4. The synthetic procedures are described in the Supplementary Material section. Formulations and gelation times are reported in Tables S1–S4. Acetone was used in order to relate the properties of aerogel powders with those of monoliths from the literature [67]. Propylene carbonate was used because it is not miscible with hexane, thereby it can be used for suspension and emulsion gelation. In propylene carbonate the release of CO_2_ was vigorous; therefore, well-shaped monoliths could not be obtained. The concentration of the monomer (Desmodur N3300) was varied between 2.75 and 11% w/w. Wet-powders were dried with supercritical fluid (SCF) CO_2_ in an autoclave to provide aerogel powders or granules.

**Figure 5:**
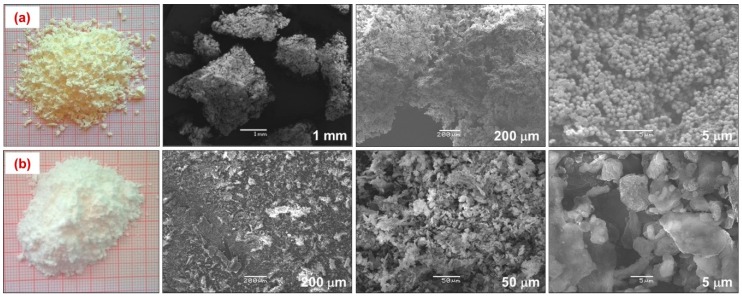
Photographs and SEM images at different magnifications of PUA powders prepared from Desmodur N3300 and water in propylene carbonate (**a**) or acetone (**b**) via vigorous agitation of the sol (monomer concentration: 5.5% w/w). Scale lengths are indicated in the right-bottom corner of each frame.

For the same monomer concentration, powders obtained from propylene carbonate sols via vigorous agitation consisted of larger particles (on the order of 1 mm; Figure 5a) than those obtained from acetone (on the order of a few hundred microns or smaller; Figure 5b). Aerogel powders were characterized in comparison to corresponding monolithic aerogels in terms of skeletal and bulk densities and BET surface areas (determined with N_2_-sorption porosimetry; Table 1). The skeletal densities were in general somewhat higher for samples prepared in propylene carbonate. The BET surface areas were consistently 2–3 times higher in powders prepared in acetone versus powders prepared in propylene carbonate (Table 1). Average pore diameters in the range of 1.7–300 nm were all in the range of 10–30 nm for all powders. Compared to monoliths with the same monomer concentration, powders had almost identical bulk and skeletal densities, and slightly lower BET surface areas (e.g., 162 versus 222 m^2^ g^–1^ for monomer concentration 5.5% w/w). It is interesting that the tapped densities of PUA powders (Table 2) increased with increasing concentration of Desmodur N3300 in the sol. The fact that the tapped densities of PUA powders varied with the monomer concentration in the sol signifies that vigorous agitation disrupts gelation at some higher aggregate level, beyond primary and secondary particles. As noted experimentally, even larger aggregates seem to coagulate with one another upon standing into larger chunks upon standing.

**Figure 6:**
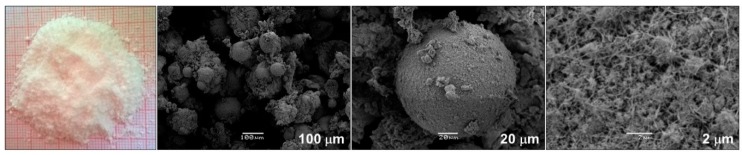
Photograph and SEM images at different magnifications of PUA powder prepared from Desmodur N3300 and water in propylene carbonate/hexane via suspension polymerization (monomer concentration: 11% w/w). Scale lengths are indicated in the right-bottom corner of each frame.

Powders were also obtained via suspension polymerization in propylene carbonate/hexane mixtures (volume ratio = 1:3). In terms of particle shape and size, suspension polymerization versus vigorous agitation gave remarkably different results: sols with low monomer concentration (5.5% w/w) under suspension polymerization gave much smaller particles (<500 microns) relative to simple vigorous agitation that gave mm-size particles (Figure 5). Higher monomer concentration sols (11% w/w) under suspension polymerization gave more-or-less uniform spherical particles, about 120 μm in diameter (Figure 6). Under higher magnification (Figure 6) the large spherical particles consisted of micron-size spherical knots, connected with fibers. Based on the fact that the BET surface area of those samples was lower than the BET surface area of the lower concentration samples (97 versus 140 m^2^ g^–1^; Table 1), it is presumed that the spherical knots shown in Figure 6 did not have an internal structure. Average pore diameters in the range of 1.7–300 nm were in the range of 20–30 nm for all powders. The skeletal (Table 1) and tapped (Table 2) densities of samples prepared via this method were similar to those obtained via the vigorous agitation method. The BET surface area of the lower concentration sample via suspension polymerization was about 3 times higher (m^2^ g^–1^) than the BET surface area of the sample via simple vigorous agitation (140 versus 52 m^2^ g^–1^; Table 1).

**Figure 7:**
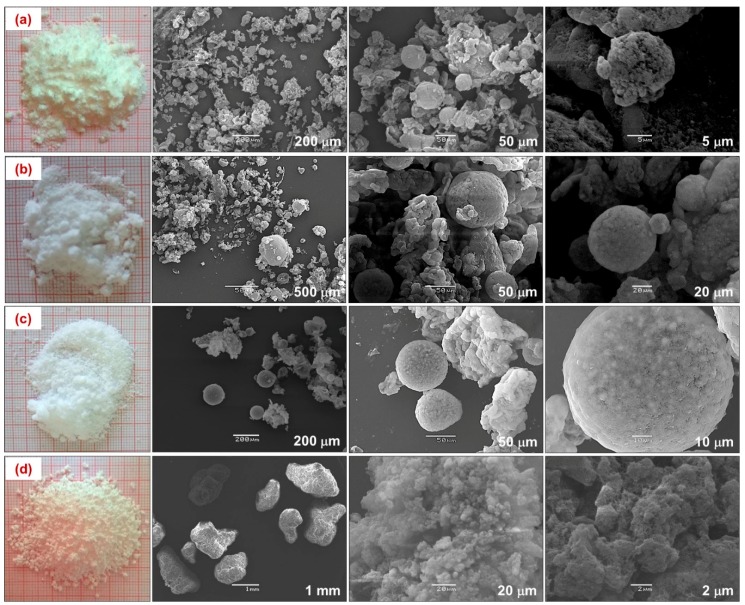
Photographs and SEM images at different magnifications of PUA powders prepared from Desmodur N3300 in propylene carbonate/hexane via emulsion polymerization. (**a**) CTAB 3.5% w/w; monomer concentration: 5.5% w/w. (**b**) CTAB 0.7% w/w; monomer concentration: 2.75% w/w. (**c**) CTAB 3.5% w/w; monomer concentration: 2.75% w/w. (**d**) NIKKOL BL-9EX 0.7% w/w; monomer concentration: 11% w/w. Scale lengths are indicated in the right-bottom corner of each frame.

The use of surfactant in powder formation was studied using two types of surfactants: CTAB (cetyltrimethylammonium bromide; Scheme 4) and NIKKOL BL-9EX (polyoxyethylene alkyl ether of lauryl alcohol; Scheme 4), at two different concentrations each: 0.7% and 3.5% w/w relative to the continuous phase. The use of CTAB as surfactant in sols with monomer concentration 5.5% w/w resulted in PUA powders of lower bulk (and tapped) density, compared with PUA samples obtained via vigorous agitation or suspension polymerization (Table 1). On the other hand, skeletal densities of all samples were almost identical (Table 1). The BET surface area was higher when the low (0.7% w/w) concentration of surfactant was used, while higher concentration (3.5% w/w) of surfactant provided the same value of BET surface area as suspension polymerization. The use of CTAB in sols with higher monomer concentrations (11% w/w) did not show any improvement in terms of densities and BET surface areas, compared to suspension polymerization (Table 1). The use of NIKKOL BL-9EX as surfactant did not affect the values of bulk and tapped density of PUA powders obtained with low monomer concertation (5.5% w/w), but PUA powders obtained with higher monomer concertation (11% w/w) were more dense than powders obtained via suspension polymerization (Table 1). The highest BET surface area (197 m^2^ g^–1^; Table 1) was measured with high surfactant concentration and low monomer concentration. The use of NIKKOL BL-9EX at higher monomer concentration sols did not show any improvement in terms of BET surface areas compared to suspension polymerization; the same observation was also made for CTAB. In terms of particle shape and size, in the presence of CTAB low monomer concentration sols gave similar particles (Figure 7a) to those obtained via suspension polymerization, while high monomer concentration sols gave bigger (on the order of 1 mm) and less uniform particles. At very low monomer concentration (2.75% w/w) most of the particles were spherical, but they could not be separated ( Figure 7b–c). In the case of NIKKOL BL-9EX, powder morphology depended on the concentration of the monomer. At lower concentrations powders were fine, smooth and fluffy, whilst at higher concentrations powders were more granular (Figure 7d). Average pore diameters in the range of 1.7–300 nm were on the order of 20–30 nm for all powders. Some recipes started showing spherical particles, but they were neither uniform nor could they be separated.

**Figure 8:**
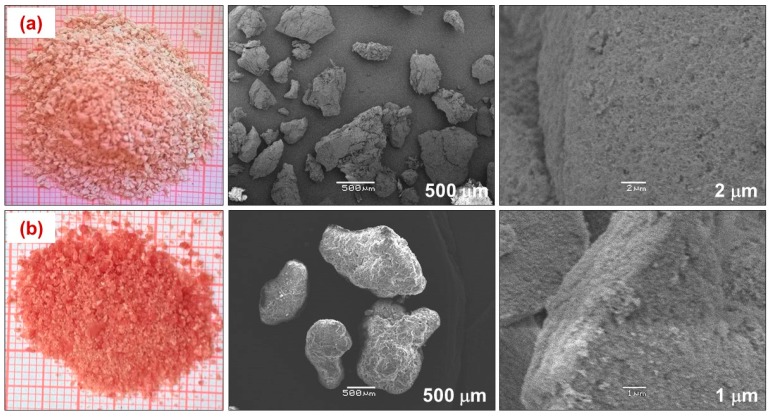
Photographs and SEM images at different magnifications of PUA powders prepared from Desmodur RE (monomer concentration: 4% w/w) and water in: (**a**) DMF via vigorous agitation of the sol and (**b**) DMF/hexane via precipitation polymerization. Scale lengths are indicated in the right-bottom corner of each frame.

PUA aerogel granules were also obtained from the reaction of aromatic Desmodur RE (Scheme 2) with water in DMF, and in the mixtures DMF/hexane and propylene carbonate/hexane (volume ratio = 1:3), using the experimental setup shown in Figure 4. The synthetic procedures are described in the Supplementary Material section. Formulations and gelation times are reported in Table S5. The concentration of the monomer (TIPM) was 4% w/w. Wet-powders were dried with SCF CO_2_ in an autoclave to provide aerogel powders, which consisted of irregular granules. Desmodur RE was used as received, i.e., 27% w/w solution of TIPM in ethyl acetate. Ethyl acetate played the role of a third solvent that rendered DMF and PC miscible with hexane. DMF/hexane, and PC/hexane mixtures here were single-systems, thereby the role of hexane was to: (a) dilute the sol; (b) decrease the solubility of the growing polymer; and, (c) induce early phase-separation of smaller particles. Thus, all powders from Desmodur RE can be classified as products of precipitation polymerization, which therefore may be considered as a variant of vigorous agitation. Aerogel powders were characterized in comparison to the corresponding monolithic aerogels in terms of skeletal density and BET surface areas (determined with N_2_-sorption porosimetry; Table 1). Bulk densities could not be measured accurately, because the materials consisted of relatively large particles of irregular shape and size. Powders obtained from DMF consisted of chunks and coarse powder Figure 8a), while those obtained from DMF/hexane were more even, consisting of particles of a more uniform size (on the order of 1 mm) and shape (Figure 8b). Powders obtained from PC/hexane consisted of bigger particles. The skeletal densities of PUA powders prepared in DMF or DMF/hexane were almost identical and very similar with the skeletal density of the monoliths (Table 1). In propylene carbonate/hexane the skeletal density was slightly higher. The BET surface areas were higher when hexane was used; the highest value was obtained in propylene carbonate/hexane (481 m^2^ g^–1^; Table 1). In all samples a 15–38% of the total surface area was assigned to micropores (Table 1), which in turn was attributed to the rigid structure of the aromatic triisocyanate—in accord with previous observations in similar materials [81,82,92]. Average pore diameters in the range of 1.7–300 nm were all found between 10 nm and 30 nm for all powders, and followed the same trend with the values obtained for the BET surface areas.

Owing to the irregular shape of the particles, the flow characteristics of the powders were assessed by calculating the percent compressibility index (100(*ρ*_t_–*ρ*_b_)/*ρ*_t_) and the Hausner ratio (*ρ*_t_/*ρ*_b_; Table 2), from bulk (*ρ*_b_) and tapped (*ρ*_t_) density data [93]. The flow characteristics of powders are classified as excellent, good, fair, passable, poor and very poor, for compressibility index values (%) in the range of 1–10, 11–15, 16–20, 21–25, 26–31 and 32–37, or Hausner ratio values in the range of 1.00–1.11, 1.12–1.18, 1.19–1.25, 1.26–1.34, 1.35–1.45 and 1.46–1.59, respectively. Overall, according to those methods the flow characteristics of most PUA aerogel powders were classified as passable, poor or very poor, except for three samples: (a) PUA powder synthesized from Desmodur RE and water in DMF/hexane showed fair flow characteristics; (b) PUA powder synthesized from Desmodur N3300 and water (monomer concentration: 11% w/w) in propylene carbonate/hexane with NIKKOL BL-9EX (0.7% w/w) showed good flow characteristics; and, (c) PUA powder synthesized from Desmodur N3300 and water (monomer concentration: 2.75% w/w) in propylene carbonate/hexane with CTAB (3.5% w/w) showed excellent flow characteristics.

### 2.2. PUA Aerogel Spherical Beads

**Figure 9:**
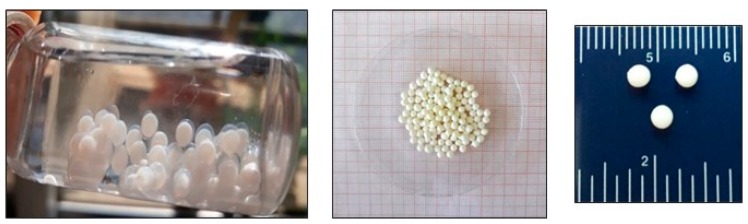
Photographs of PUA aerogel beads (wet-gels and aerogels) [94].

**Figure 10:**
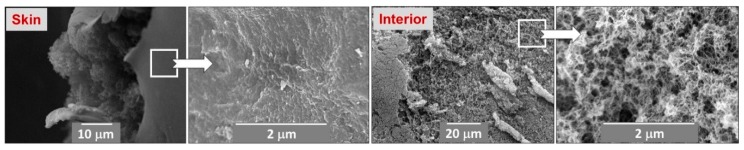
SEM images of spherical PUA aerogel beads at different magnifications: skin and interior, as indicated [94].

Spherical PUA beads were synthesized from the reaction of Desmodur N3300 (Scheme 2) (in propylene carbonate) and ethylene diamine (Scheme 3) in heavy mineral oil, via the dripping method at r.t. and SCF drying. The synthetic method is simple, cost-efficient and suitable for large-scale production of PUA aerogel beads. The beads (Figure 9) can be 2–4 mm in diameter with narrow size distributions (full width at half maximum: 0.3–0.4 mm) and they have low density (0.16 g cm^–3^), high porosity (>87% v/v), and high surface area (200 m^2^ g^–1^). Analogous monoliths had similar density and porosity, but much lower surface area (70 m^2^ g^–1^). To our knowledge this work comprises the first-time report of uniform, millimeter-size PUA particles via a method that can be easily applied for large-scale production. The material properties of those beads were similar to those of chemically related and morphologically similar monoliths reported in the literature [67] from the reaction of the same triisocyanate with water in acetone, suggesting that nanostructure rather than minor differences in chemical composition is the property-determining factor. SEM (Figure 10) showed that the surface of the beads had a different morphology from their interior, which may be explained by the diffusion profile of ethylenediamine in the propylene carbonate droplets [94]. The water contact angle in the interior of the beads (by sanding) was about 102^o^. Dividing that value by the porosity of the beads gave a ratio of about 1.2. That ratio is referred to as the K-index, which was introduced recently as a means to quantify nanomorphology [91]. The development of the *K*-index was based on a vast array of monolithic polyurea aerogels, and therefore it applies directly to the nanomorphology of the PUA beads. High aspect ratio (caterpillar-like) nanostructures are expected to have *K*-index = 1.2, in agreement with both the SEM of PUA beads (Figure 10) and the ratio of the experimental contact angle of water droplets (102^o^) over the porosity of the beads (87% v/v).

## 3. Polyimide (PI) Aerogels

**Scheme 5:**
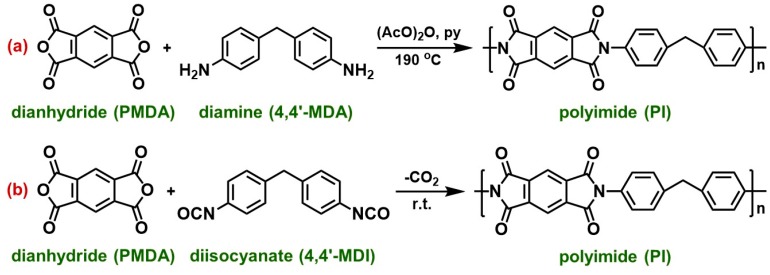
Synthesis of chemically identical polyimide (PI) aerogels via two different routes: (**a**) from a dianhydride and a diamine (the DuPont route), (**b**) from the same dianhydride and the corresponding diisocyanate to the diamine of part (a). PMDA: pyromellitic dianhydride; 4,4′-MDA: 4,4′-diaminodiphenylmethane; and, 4,4′-MDI: 4,4′-methylenebis(phenyl isocyanate).

Polyimides (PI) are a class of engineering plastics, and are utilized for high temperature applications. PIs are synthesized commercially by two methods: (a) condensation (>190 °C) of aromatic dianhydrides and amines (the DuPont route; Scheme 5a) [95], and (b) crosslinking (>300 °C) of norbornene end-capped imide oligomers (referred to as the PMR route; PMR: polymerization of monomer reactants) [96,97]. PI aerogels based on the DuPont route were reported in a 2006 US patent [70]. An alternative method for the synthesis of PI aerogels was reported in 2010. That method was based on the reaction of aromatic dianhydrides with multifunctional isocyanates at room temperature [71,73] (Scheme 5b). Both the DuPont route and the isocyanate route yield chemically identical products. The main advantages of the isocyanate route are: the low cost of the isocyanates, no sacrificial reagents needed (versus acetic anhydride and pyridine by the DuPont route) needed, and the only byproduct is CO_2_ (versus HCl from the DuPont route). Detailed structural analysis with small angle neutron scattering (SANS) has revealed that materials synthesized in *N*-methyl pyrrolidone (NMP) via either route consist of similar size primary and secondary particles; SEM, however, has shown that secondary particles assembled differently: into fibers in the isocyanate route, and into globular aggregates in the amine route [70].

### 3.1. Particulate PI Aerogels

**Figure 11:**
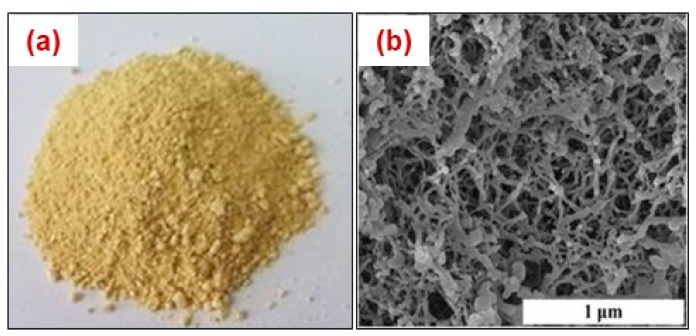
Photograph (**a**) and SEM image (**b**) of PI aerogel powders obtained from the imidization of PMDA/4,4′-MDA-derived PAA. Adapted by permission from Springer Nature Customer Service Centre GmbH: Ref. [98].

Nanofibrous PI powders (Figure 11) have been produced by mechanical agitation of a gel-like poly(amic acid) (PAA), which was obtained from pyromellitic dianhydride (PMDA; Scheme 5) and 4,4′-methylenedianiline (4,4′-MDA; Scheme 5) in THF/MeOH mixed solvent [98]. Interestingly, rather than a real gel (i.e., an elastic solid), the gel-like PAA product was a clear glass-like viscous solution that, upon addition of acetone, phase-separated into an opaque-white wet-gel. The authors pointed out that a gel-like PAA product was obtained only from the specific monomers; other starting materials yielded only viscous solutions of the corresponding PAAs. Subsequently, acetone was solvent-exchanged with cyclohexane, which was removed under vacuum at about 10 °C yielding PAA aerogels that were imidized by heating successively at 100, 200 and finally at 300 °C. If addition of acetone into the gel-like PAA product was accompanied by vigorous mechanical stirring, the end product was an aerogel powder rather than a monolith. In contrast to the report of Kong et al. on PUA powders (see above) [90], vigorous agitation did not disrupt the microfibrous network structure of the PI, which was identical in both monoliths and powders. This new, green, scalable and economic process provided PI aerogel particles with nanofibrous morphology, low density (0.175 g cm^–1^) and high thermal stability. Their decomposition temperatures were above 500 °C.

**Figure 12:**
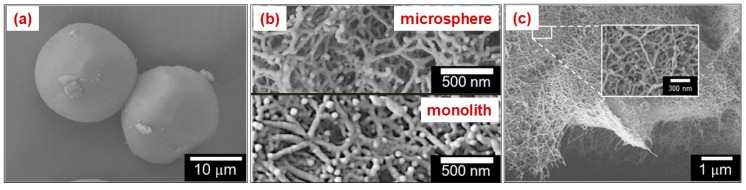
SEM images of: (**a**) PI aerogel microparticles, (**b**) their skeletal networks in comparison with corresponding monoliths, and (**c**) the surface of PI aerogel microparticles. Adapted with permission from Ref. [54]. Copyright 2016 American Chemical Society.

PI aerogel particles have been prepared in DMF/cyclohexane emulsions [54]. A solution consisting of PAA oligomers was prepared by dissolving PMDA and 2,2′-dimethylbenzidine (DMBZ; Scheme 3) in DMF at r.t. 1,3,5-Triaminophenoxybenzene (TAB; Scheme 3) was added to the sol, followed by drop-wise addition of acetic anhydride and pyridine. The resulting PI sol was dispersed in cyclohexane, using Span 85 and Hypermer 1599 as surfactants. The microparticles were washed with acetone and dried using SCF CO_2_. The resulting PI aerogel particles had mean diameters of 40.0 μm, pore volumes of 3.38 cm^3^ g^–1^, and a fibrous internal microstructure, similar to the one observed in the corresponding aerogel monoliths (Figure 12b). Their BET surface areas were lower than those of the monoliths (512 versus 717 m^2^ g^–1^), which was attributed to the shorter aging period.

### 3.2. PI Aerogel Spherical Beads

**Figure 13:**
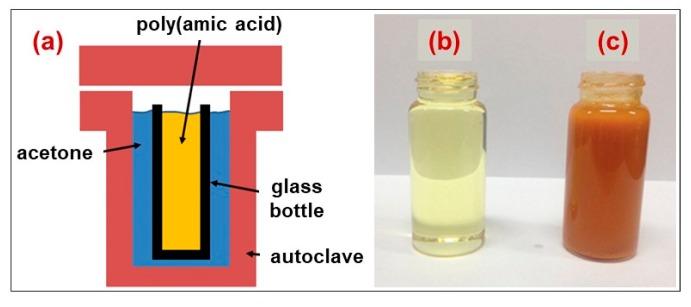
(**a**) Setup for the fabrication of PI aerogel microparticles. (**b**) Glass bottle with PAA before curing. (**c**) PI aerogel microparticles after curing. Copyright (2014) Wiley. Used with permission from Ref. [99].

**Figure 14:**
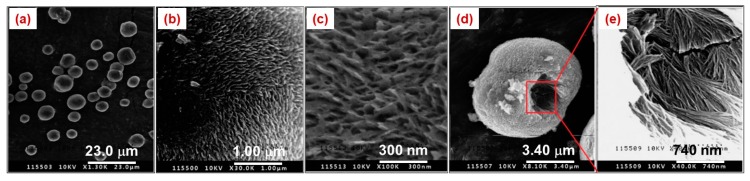
SEM images of: (**a**) PI aerogel microparticles; (**b**,**c**) their surfaces; (**d**,**e**) their interior. Copyright (2014) Wiley. Used with permission from Ref. [99].

**Figure 15:**
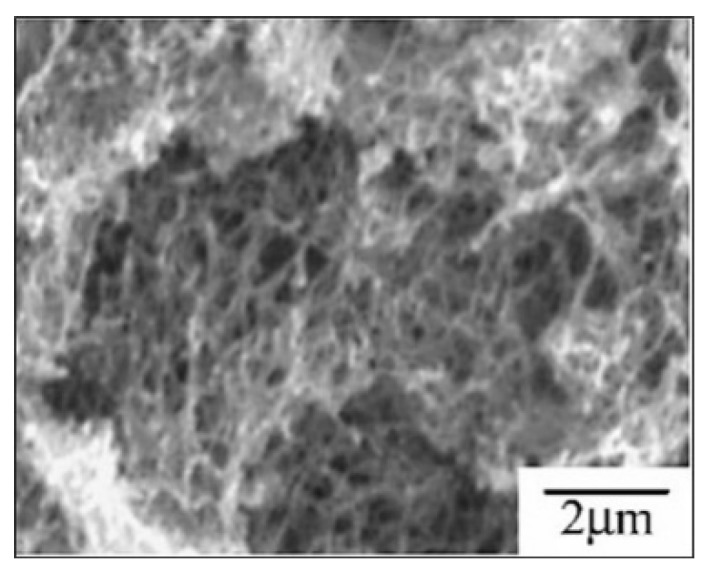
SEM image of the interior of a PI aerogel monolith derived from PMDA, 4,4′-ODA and TAPB. Copyright (2007) Wiley. Used with permission from Ref. [100].

Millimeter-size PI aerogel particles have been synthesized using the experimental setup shown in Figure 13 [99]. PAAs were synthesized via polymerization of PMDA and 4,4′-oxydianiline (4,4′-ODA; Scheme 3) in NMP, and were transformed to PI spherical microparticles by gradually heating the solution at 250 °C in an autoclave (Figure 13). Acetone was used to fill the space between the autoclave and the reaction vessel. Under the conditions of the reaction (temperature of 250 °C and pressure above 4.66 MPa), acetone is a supercritical fluid, and its high pressure prevented NMP from evaporating. The particles (Figure 14) were spherical with diameters ranging from 3.6 to 4.9 μm, BET surface areas of 103 m^2^ g^–1^, porosities of 80% v/v, typical pore sizes at around 15 nm (Figure 14), and good thermal stability (>500 °C). Qualitatively, they also showed good oil adsorption capacity. Analogous PI aerogel monoliths reported were synthesized from PMDA and 4,4′-ODA, with 1,3,5-tris(4-aminophenyl)benzene (TAPB) as crosslinker, in NMP. Morphologically, the internal structure of those monoliths was more fibrous than the internal structure of spherical particles (Figure 15) [100].

## 4. Phenolic Resin Aerogels (Including Polybenzoxazines)

Resorcinol-formaldehyde (RF) aerogels and polybenzoxazine (PBO) aerogels are the main carbon aerogel precursors [14,56,58,60].

**Scheme 6:**
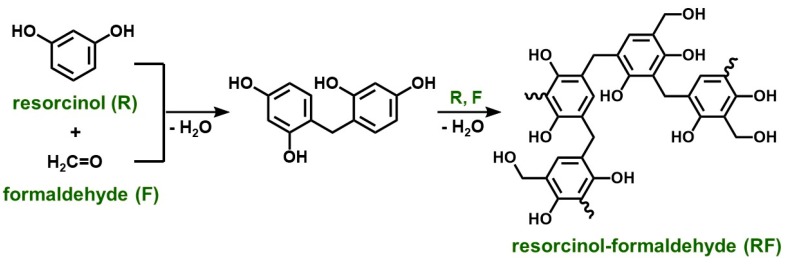
**Scheme 6**. Synthesis of RF aerogels.

RF aerogels were first prepared in 1989 via base-catalyzed gelation of aqueous solutions of resorcinol and formaldehyde (up to 7 days at 85 °C), followed by aging (>3 days in CF_3_COOH at 45 °C) and drying from SCF CO_2_ [56,57,58,59]. Acid-catalyzed processes have also been reported (Scheme 6) and include reactions in: (a) HClO_4_/acetone at 45 °C followed by 3 days of aging [101], (b) aqueous HNO_3_ at 80 °C (2 day gelation, 7 days aging) [102], (c) acetic acid at room temperature (1 day gelation, aging for 3 days at 50 °C and 3 days at 90 °C) [103,104], and (d) HCl/CH_3_CN at 80 °C (gelation within minutes, no aging) [55]. RF aerogels obtained by the acid-catalyzed routes are chemically similar to the ones obtained via the base-catalyzed aqueous gelation process.

**Scheme 7:**
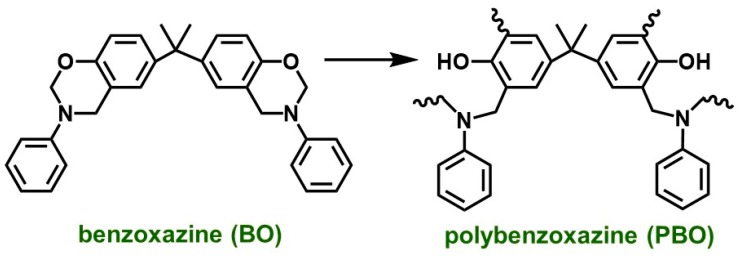
Synthesis of PBO aerogels via polymerization of a BO monomer derived from the reaction of bisphenol A, aniline, and formaldehyde.

Polybenzoxazines (PBOs) are phenolic resins with high mechanical strength, inherent flame retardancy, low water retention, exceptional thermal properties (high glass transition and decomposition temperatures), and high carbonization yields [105]. Although benzoxazines were known since 1944 [106], the systematic study of their polymers began 50 years later [107]. PBOs are typically prepared via thermally induced ring-opening polymerization of suitable benzoxazine (BO) monomers, with most known the condensation product of bisphenol A, aniline, and formaldehyde (Scheme 7) [107,108,109,110]. Monolithic PBO aerogels have been synthesized via polymerization of that monomer in xylene at 130 °C for 96 h (heat-induced polymerization) [62,63], or in DMF at room temperature for a few hours (HCl-catalyzed polymerization) [64]. The HCl-catalyzed polymerization imposed additional crosslinking that resulted in higher surface areas and lower thermal conductivity.

### 4.1. RF Aerogel Beads

RF aerogel spherical particles have been synthesized by various methods: (a) emulsion polymerization in water/cyclohexane, using K_2_CO_3_ as basic catalyst and a surfactant of the non-ionic sorbitan alkyl ester series by NOF Corp, followed by solvent exchange with acetone and drying from SCF CO_2_ [48], (b) inverse suspension polymerization in water/peanut oil, with Na_2_CO_3_ as catalyst, followed by solvent exchange with alcohol and drying under alcohol supercritical conditions [49], and (c) an injection emulsification method, in which a syringe pump was used to inject droplets of an aqueous solution, containing resorcinol, formaldehyde and Na_2_CO_3_, into the continuous phase of cyclohexane, containing Span 80 as emulsifier, followed by solvent exchange with acetone and drying from SCF CO_2_ [53]. However, in all those cases, the RF aerogel particles were not characterized, as the authors were more interested in the products of their pyrolysis.

**Figure 16:**
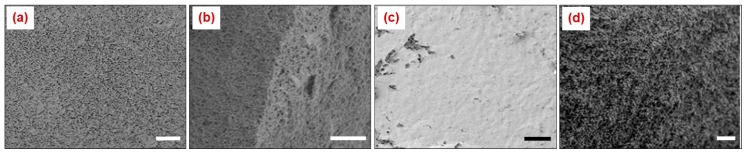
SEM images of outer surfaces (**a**,**c**) and inner surfaces of skins and bulk morphologies (**b**,**d**) of RF foam aerogel beads obtained via reactions catalyzed by triethylamine (a, b: 0.39% w/w), or tri(n-butyl)amine (c, d: 0.039% w/w). Scale bar: 2 μm. Reprinted by permission of Taylor & Francis Ltd., http://www.tandfonline.com, Ref. [111].

RF aerogel foam beads, with diameter of 1 mm and a smooth membrane covering their surface (Figure 16) have been synthesized by dropwise addition of the aqueous resorcinol/formaldehyde/Na_2_CO_3_ solution in a CCl_4_/mineral oil mixture, which had equal density to the RF sol and also contained a basic phase-transfer catalyst (triethylamine or tri(*n*-butyl)amine) [111]. After the droplets gelled, they were solvent exchanged with 2-propanol and underwent SCF CO_2_ drying. Those aerogels had densities in the range of 104–184 mg cm^−3^, increasing with decreased concentration of the catalyst and decreased length of its alkyl chain. The cell size of the foams (from SEM images) ranged from 55 to 93 nm when trimethylamine was used and from 40 to 46 nm when tri(*n*-butyl)amine was used; in both cases, cell sizes increased with the catalyst concentration. In analogy to what has been observed with PUA beads [94] (Figure 10), the morphology of the surface of the RF aerogel beads was smoother (especially when tri(*n*-butyl)amine was used as catalyst), while internally all RF beads had more or less the same structure (Figure 16).

### 4.2. PBO Aerogel Beads

**Figure 17:**
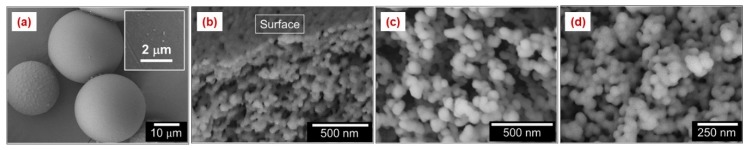
SEM image of: (**a**) PBO aerogel microparticles (inset: surface of the sphere), (**b**) fractured surface of PBZ aerogel microparticles, and (**c**,**d**) ensemble of spherical polybenzoxazine domains that formed the internal structure of aerogel microparticles and monolith, respectively. Adapted with permission from Ref. [54]. Copyright 2016 American Chemical Society.

There is only one example in the literature for particulate PBO aerogels. Reported fabrication of PBO aerogel particles from the reaction of 4,4′-isopropylidenediphenol, aniline and paraformaldehyde catalyzed by HCl [54]. The synthetic process was similar to the one described for PI aerogel powders derived from PMDA, DMBZ and TAB, as described in the previous section [54]. Compared to PIs, PBO particles had a more regular spherical shape, particulate (instead of fibrous) microstructure (Figure 17) and lower BET surface areas, which did not differ significantly to the ones of the corresponding monoliths (55.4–57.8 m^2^ g^–1^). However, PBO microparticles had twice the pore volume in the 1.7–300 nm range than the corresponding monoliths (0.20 versus 0.10 cm^3^ g^−1^), which was attributed to lower shrinkage. The mean diameter of PBO microspheres was 32.7 μm and they had a 100 nm thick dense skin layer. SEM images showed that the internal structure of PBO microparticles consisted of smaller spheres itself.

## 5. Conclusions

Despite the vast literature on monolithic aerogels derived from synthetic polymers, including aerogels based on phenolic resins (e.g., resorcinol-formaldehyde and polybenzoxazines), polyureas, polyimides, polyamides, polyurethanes, and ROMP-derived aerogels, including polynorbornene and polydicyclopentadiene, only a few examples of synthetic polymer aerogels in particulate form have been described in the literature up to date, and have therefore been the subject of this mini-review. Aerogels classified as particulate include materials in the form of powders, irregular granules and spherical beads. Polymeric aerogels prepared in those forms include mostly polyurea aerogels derived from the reaction of isocyanates with amines or water under four different conditions: (a) disruption of gelation by vigorous agitation of the sol (powders and granules), (b) addition of non-solvents in the sol (powders and granules), (c) emulsion polymerization with surfactants (powders, granules. microspheres), and (d) dripping of the sol in a non-solvent (millimeter-size beads). Polyimide aerogels have also been synthesized from pyromellitic dianhydride and amines via (a) vigorous agitation of the sol (powders), (b) emulsion polymerization (powders), and (c) by mixing of polyamic acid sols with acetone as a supercritical fluid (spherical particles). Finally, phenolic type aerogels from resorcinol-formaldehyde and polybenzoxazine have been prepared in particulate form (beads) via emulsion and suspension polymerization processes, as well as with the dripping method. Table 3 summarizes all methods that have been utilized for the preparation of particulate aerogels from synthetic polymers. Manufacturing aerogels in a particulate rather than a monolithic form renders the bulk product rather insensitive to defects (e.g., cracks), reduces processing times, broadens the horizon of possible applications (as adsorbers, desiccants, additives for foods, cosmetics, construction materials, vehicles for drug delivery, etc.) via the one size fits all approach, and overall brings aerogels closer to consumer products. With respect to the most well-established application of aerogels as thermal insulators, the empty space between the particles compromises thermal conductivity. A solution to that problem will involve the packing of multi-dispersed particles.

## Figures and Tables

**Table 1:** Selected properties of particulate PUA aerogels.

Sample I.D.	Particle Diameter	Bulk Density *ρ*_b_ (g cm^–3^)		Skeletal Density *ρ*_s_ (g cm^–3^)		BET Surf. Area *σ* (m^2^ g^–1^) [Micropore Surf. Area]^a^		Particle Radius^b^ (nm)	
		Particle	Monolith	Particle	Monolith	Particle	Monolith	Particle	Monolith
N3300-H_2_O 2.75% acetone	<20 μm	0.023 ± 0.001	0.028 ± 0.002	1.35 ± 0.02	1.31 ± 0.06	162 ± 9	159 ± 8	13.7 ± 0.8	14.4 ± 1.0
N3300-H_2_O 5.5% acetone	<20 μm	0.048 ± 0.001	0.07 ± 0.01	1.182 ± 0.006	1.20 ± 0.01	162 ± 8	222 ± 13	15.7 ± 0.8	11.3 ± 0.7
N3300-H_2_O 11% acetone	<50 μm	0.113 ± 0.008	0.126 ± 0.001	1.166 ± 0.007	1.19 ± 0.01	113 ± 7	165 ± 9	22.8 ± 1.4	15.3 ± 0.8
N3300-H_2_O 2.75% PC	1–2 mm	0.036 ± 0.001	^c^	1.39 ± 0.03	^c^	95 ± 5	^c^	22.7 ± 1.3	^c^
N3300-H_2_O 5.5% PC	1–2 mm	0.058 ± 0.001	^c^	1.37 ± 0.03	^c^	52 ± 3	^c^	42.1 ± 2.6	^c^
N3300-H_2_O 11% PC	-	^d^	^c^	^d^	^c^	^d^	^c^	^d^	^c^
N3300-H_2_O 5.5% PC/hexane	<500 μm	0.060 ± 0.001		1.36 ± 0.03		140 ± 7		15.8 ± 0.9	
N3300-H_2_O 11% PC/hexane	120 μm	0.034 ± 0.001		1.29 ± 0.02		97 ± 6		24.0 ± 1.5	
N3300-H_2_O 2.75% CTAB 0.7%	<200 μm	0.045 ± 0.003		1.24 ± 0.01		141 ± 8		17.2 ± 1.0	
N3300-H_2_O 5.5% CTAB 0.7%	100–200 μm	0.025 ± 0.002		1.38 ± 0.04		174 ± 10		12.5 ± 0.8	
N3300-H_2_O 11% CTAB 0.7%	~1 mm	0.033 ± 0.001		1.32 ± 0.02		80 ± 4		28.4 ± 1.5	
N3300-H_2_O 2.75% CTAB 3.5%	<200 μm	0.057 ± 0.001		1.29 ± 0.01		162 ± 9		14.4 ± 0.8	
N3300-H_2_O 5.5% CTAB 3.5%	200–250 μm	0.038 ± 0.003		1.28 ± 0.01		144 ± 8		16.3 ± 0.9	
N3300-H_2_O 11% CTAB 3.5%	~1 mm	0.035 ± 0.002		1.29 ± 0.01		51 ± 3		45.6 ± 2.7	
N3300-H_2_O 5.5% NIKKOL BL-9EX 0.7%	<100 μm	0.078 ± 0.002		1.66 ± 0.08		4 ± 1		452 ± 115	
N3300-H_2_O 11% NIKKOL BL-9EX 0.7%	~1 mm	0.094 ± 0.001		1.41 ± 0.03		85 ± 5		25.0 ± 1.6	
N3300-H_2_O 5.5% NIKKOL BL-9EX 3.5%	~100 μm	0.052 ± 0.003		1.47 ± 0.06		197 ± 11		10.4 ± 0.7	
N3300-H_2_O 11% NIKKOL BL-9EX 3.5%	0.5–1 mm	0.077 ± 0.001		1.50 ± 0.07		76 ± 4		26.3 ± 1.9	
RE-H_2_O 4% DMF	~500 μm	^e^	0.393 ± 0.033	1.254 ± 0.001	1.353 ± 0.009	215 ± 12 [15]	205 ± 11 [20]	11.1 ± 0.6	10.8 ± 0.6
RE-H_2_O 4% DMF/hexane	~1 mm	0.30 ± 0.01		1.264 ± 0.001		394 ± 21 [43]		6.0 ± 0.3	
RE-H_2_O 4% PC/hexane	~1 mm	^e^		1.324 ± 0.008		481 ± 24 [22]		4.7 ± 0.2	
N3300-EDA 11% [94]	2.68 ± 0.16 mm	0.166 ± 0.001	0.110 ± 0.001	1.246 ± 0.005	1.236 ± 0.006	197 ± 12	73 ± 4	12.2 ± 0.7	33.2 ± 1.8

^a^ Micropore surface area via *t*-plot analysis, according to the Harkins and Jura. ^b^ Calculated *via*: *r* = 3/(*ρ*_s_ × *σ*). ^c^ Monoliths of irregular shape. ^d^ Owing to fast reaction the entire sol gelled and subsequently the gel was broken by the mechanical stirrer into large monolithic pieces. ^e^ Small amount of powder, not sufficient for this measurement. Most of the sample was in the form of large chunks and lumps. PC: propylene carbonate.

**Table 2:** Flow characteristics of particulate PUA aerogels.

Sample I.D.	Bulk Density *ρ*_b_ (g cm^–3^)	Tapped Density *ρ*_t_ (g cm^–3^)	Compressibility Index ^a^	Hausner Ratio ^b^	Flow Quality
N3300-H_2_O 2.75% acetone	0.023 ± 0.001	0.036 ± 0.001	36	1.57	very poor
N3300-H_2_O 5.5% acetone	0.048 ± 0.001	0.063 ± 0.005	24	1.31	passable
N3300-H_2_O 11% acetone	0.113 ± 0.008	0.166 ± 0.008	32	1.47	very poor
N3300-H_2_O 2.75% PC	0.036 ± 0.001	0.047 ± 0.001	23	1.31	passable
N3300-H_2_O 5.5% PC	0.058 ± 0.001	0.078 ± 0.002	26	1.34	passable
N3300-H_2_O 5.5% PC/hexane	0.060 ± 0.001	0.078 ± 0.001	23	1.30	passable
N3300-H_2_O 11% PC/hexane	0.034 ± 0.001	0.050 ± 0.001	32	1.47	very poor
N3300-H_2_O 2.75% CTAB 0.7%	0.045 ± 0.003	0.068 ± 0.001	34	1.51	very poor
N3300-H_2_O 5.5% CTAB 0.7%	0.025 ± 0.002	0.038 ± 0.001	34	1.52	very poor
N3300-H_2_O 11% CTAB 0.7%	0.033 ± 0.001	0.045 ± 0.001	27	1.36	poor
N3300-H_2_O 2.75% CTAB 3.5%	0.057 ± 0.001	0.059 ± 0.002	3	1.04	excellent
N3300-H_2_O 5.5% CTAB 3.5%	0.038 ± 0.003	0.059 ± 0.005	36	1.55	very poor
N3300-H_2_O 11% CTAB 3.5%	0.035 ± 0.002	0.050 ± 0.003	30	1.43	poor
N3300-H_2_O 5.5% NIKKOL BL-9EX 0.7%	0.078 ± 0.002	0.104 ± 0.004	25	1.33	passable
N3300-H_2_O 11% NIKKOL BL-9EX 0.7%	0.094 ± 0.001	0.107 ± 0.003	12	1.14	good
N3300-H_2_O 5.5% NIKKOL BL-9EX 0.7%	0.052 ± 0.003	0.074 ± 0.003	30	1.42	poor
N3300-H_2_O 11% NIKKOL BL-9EX 0.7%	0.077 ± 0.001	0.097 ± 0.002	21	1.26	passable
RE-H_2_O 4% DMF/hexane	0.30 ± 0.01	0.36 ± 0.03	17	1.20	fair

^a^ Compressibility index: 100(*ρ*_t_ − *ρ*_b_)/*ρ*_t_. ^b^ Hausner ratio: *ρ*_t_/*ρ*_b_; *ρ*_b_: bulk density, *ρ*_t_: tapped density.

**Table 3:** Summary of the synthetic methods used for the preparation of aerogel particles from synthetic polymers reviewed in this work.

Type of Aerogel	Synthetic Method	References
Polyurea (PUA)	Precipitation polymerization	[88,89,90]
	Vigorous agitation	this work
	Suspension polymerization	this work
	Emulsion polymerization	this work
	Dripping method	[94]
Polyimide (PI)	Mechanical agitation	[98]
	Emulsion polymerization	[54]
	Heating in an autoclave	[99]
Resorcinol-formaldehyde (RF)	Emulsion polymerization	[48]
	Inverse suspension polymerization	[49]
	Injection emulsification	[53]
	Dripping method	[111]
Polybenzoxazine (PBO)	Emulsion polymerization	[54]

## Supplementary Materials

The following are available online at https://www.mdpi.com/1996-1944/12/9/1543/s1: Experimental Section (1. Materials; 2. Methods and Equipment; 3. Synthetic Procedures), Table S1: Formulations of PUA powders from Desmodur N3300 for preparation in acetone via Vigorous Agitation, Table S2: Formulations of PUA powders from Desmodur N3300 for preparation in PC via Vigorous Agitation, Table S3: Formulations of PUA powders from Desmodur N3300 for preparation in PC/hexane via Suspension Polymerization, Table S4: Formulations of PUA powders from Desmodur N3300 for preparation in PC/hexane via Emulsion Polymerization, Table S5: Formulations of PUA powders from Desmodur RE for preparation via Vigorous Agitation or via Precipitation Polymerization.
