# Supplementary material for: Synthetic Polymer Aerogels in Particulate Form
Source: Materials (Basel). 2019 May 10;12(9):1543. doi: 10.3390/ma12091543 (PMC6539448; doi:10.3390/ma12091543)
Supplement: Supplementary file 1 [file materials-12-01543-s001.pdf]

materials

Supplementary Material
Synthetic Polymer Aerogels in Particulate Form

Patrina Paraskevopoulou *, Despoina Chriti, Grigorios Raptopoulos and George C. Anyfantis

Laboratory of Inorganic Chemistry, Department of Chemistry, National and Kapodistrian University of Athens, Panepistimiopolis Zografou, Athens 15771, Greece; paraskevopoulou@chem.uoa.gr (P.P.); chritides@chem.uoa.gr (D.C.); grigorisrap@chem.uoa.gr (G.R.); gc.anyfantis@gmail.com (G.C.A.)

* Correspondence: paraskevopoulou@chem.uoa.gr (P.P.); Tel.: +30-210-727-4381; Fax: +30-210-727-4782

Experimental Section

1. Materials.

Monomers Desmodur N3300 and Desmodur RE triisocyanates were kindly provided from Covestro. Desmodur N3300 was supplied in neat form and Desmodur RE was supplied as a solution in ethyl acetate. Both monomers were used as received. NIKKOL BL-9EX (Polyoxyethylene alkyl ether of lauryl alcohol) was kindly provided from Nikko Chemicals Co., Ltd. and hexadecyltrimethylammonium bromide (CTAB, 99+% pure) was purchased from ACROS. Both surfactants were used as received. Anhydrous acetone was produced from lower grade solvent by distilling over P_2O_5. *N*,*N*-Dimethylformamide (DMF, 99.8% extra dry) and propylene carbonate (PC, 99.5% pure) were purchased from ACROS and were used as received. Triethylamine (99% pure) was purchased from ACROS and was distilled before use.

2. Methods and Equipment.

Supercritical fluid (SCF) drying was carried out in an autoclave (E3100, Quorum Technologies, East Sussex, UK). Wet-gels were placed in the autoclave at 12 °C and were covered with acetone. Liquid CO_2 was allowed in the autoclave; acetone was drained out as it was being displaced by liquid CO2 (5×; 1 per 30 min). Afterwards, the temperature of the autoclave was raised to 45 °C and was maintained for 1 h. Finally, the pressure was gradually released, allowing SCF CO_2 to escape as a gas, leaving dry-gels (aerogels). Bulk densities (ρ_b) of monoliths were determined from their mass and volume. Bulk densities of powders were calculated using a 1 mL volumetric flask, which was filled with powder and then weighed. The powder in the flask was packed under its own weight. Tapped densities (ρ_t) of powders were determined after the powder in the flask was packed under its own weight and then tapped. The powder settled and the volume created was filled with more powder packed under its own weight. The flask was tapped again, more powder was added and the cycle was repeated until the flask was filled with settled powder up to the mark. N_2-sorption measurements were made on a Micromeritics Tristar II 3020 surface area and porosity analyzer (Micromeritics, Norcross, GA, USA). Skeletal densities (ρ_s) were determined by He pycnometry, using a Micromeritics AccuPyc II 1340 pycnometer (Micromeritics, Norcross, GA, USA). SEM studies were carried out on a Scanning Electron Microscopy (SEM) JEOL JSM-5600. The system was operating at 20 KV, 0.5 nA and 50 sec time of analysis.

3. Synthetic Procedures

3.1. Synthesis of Polyurea (PUA) Powders from Desmodur N3300

Vigorous agitation in acetone or PC. All formulations are shown in Tables S1 and S2. Desmodur N3300 was dissolved in the solvent of choice in a round bottom flask equipped with a mechanical stirrer, set at a stirring rate of 250-300 rpm. Subsequently, water and then triethylamine were added to the flask. The resulting solution was stirred at room temperature. After the end of the reaction the

solution became cloudy and was left to stir with constant rotation speed for two times the reaction time. In order to remove solvent, solution was transferred into centrifuge tubes. Wet powders were washed with acetone followed by centrifugation for 10 min. This procedure was carried out 5 times for washing powders. The washed powders were dried with liquid CO_2 in an autoclave taken out as supercritical fluid (SCF).

Suspension polymerization in PC/hexane. All formulations are shown in Table S3. Desmodur N3300 was dissolved in PC in a round bottom flask equipped with a mechanical stirrer and set at a stirring rate of 250-300 rpm. Subsequently, water and then triethylamine were added to the flask. The resulting solution was stirred at room temperature. After the end of the reaction the solution became cloudy and was stirred with constant rotation speed for two times the reaction time. In order to remove solvent, solution was transferred into centrifuge tubes. Wet powders were washed with acetone followed by centrifugation for 10 min. This procedure was carried out 5 times for washing powders. The washed powders were dried with liquid CO_2 in an autoclave taken out as SCF.

Emulsion polymerization in PC/hexane. All formulations are shown in Table S4. Two solutions were prepared, one containing the surfactant of choice in hexane (continuous phase) and the second one containing the dispersed phase. The latter was prepared by dissolving Desmodur N3300 in PC and subsequently adding water and triethylamine as catalyst. To avoid the reaction starting before mixing the two phases, the dispersed phase was prepared in an ice bath. The dispersed phase was added to the continuous one in a round bottom flask. The resulting mixture was stirred at 400 rpm, at room temperature. After the end of the reaction the solution was transferred into centrifuge tubes. Wet particles were washed with acetone followed by centrifugation for 10 min. This procedure was carried out 5 times for washing powders. The washed powders were dried with liquid CO_2 in an autoclave taken out as SCF.

3.2. Synthesis of PUA Powders from Desmodur RE

Vigorous agitation in DMF. Desmodur RE (the quantity of TIPM in Desmodur RE was calculated based on the 27% w/w concentration given by the supplier) was dissolved in DMF in a round bottom flask equipped with a mechanical stirrer and set at a stirring rate of 250-300 rpm. Subsequently, water and then triethylamine was added to the flask. The resulting solution was stirred at room temperature. After the end of the reaction the solution became viscous and was stirred with constant rotation speed for 2x the reaction time. In order to remove solvent, solution was transferred into centrifuge tubes. Wet powders were washed with acetone followed by centrifugation for 10 min. This procedure was carried out 5 times for washing powders. The washed powders were dried with liquid CO_2 in an autoclave taken out as SCF.

Precipitation polymerization in DMF/hexane or PC/hexane. All formulations are shown in Table S5. Desmodur RE (the quantity of TIPM in Desmodur RE was calculated based on the 27% w/w concentration given by the supplier) was added with the solvent of choice in a round bottom flask, equipped with a mechanical stirrer and set at a stirring rate of 250-300 rpm. Subsequently, water and then triethylamine were added to the flask. The resulting solution was stirred at room temperature. After the end of the reaction the solution became viscous and was stirred with constant rotation speed for two times the reaction time. In order to remove solvent, solution was transferred into centrifuge tubes. Wet powders were washed with acetone followed by centrifugation for 10 min. This procedure was carried out 5 times for washing powders. The washed powders were dried with liquid CO_2 in an autoclave taken out as SCF.

3.3. Synthesis of PUA Monolithic Aerogels

Monolithic aerogels were synthesized using the same formulations as for powders, by pouring sols in suitable moulds.

Table S1. Formulations of PUA powders from Desmodur N3300 for preparation in acetone via Vigorous Agitation. [a,b]

[N3300]		N3300		H_2O		Et_3N			Acetone	Total Volume	Gelation Time
(% w/w)	(M)	(g)	(mol)	(mL)	(mol)	(mL)	(mol)	(%w/w)	(mL)	(mL)	(min)
2.75	0.06	5.5	0.011	0.6	0.033	2.1	0.015	1.0	188	195.4	570
5.5	0.11	11	0.022	1.2	0.067	4.2	0.030	1.9	188	202.8	180
11	0.20	22	0.044	2.4	0.133	8.4	0.060	3.4	188	217.6	30
16.5	0.28	33	0.065	3.6	0.200	12.6	0.090	4.7	188	232.4	4

[a] The resulting solution was stirred at room temperature (stirring rate 250–300 rpm). [b] Solvent was removed by centrifugation for 10 min (4000 rpm).

Table S2. Formulations of PUA powders from Desmodur N3300 for preparation in PC via Vigorous Agitation. [a,b]

[N3300]		N3300		H_2O		Et_3N			PC	Total Volume	Gelation Time
(% w/w)	(M)	(g)	(mol)	(mL)	(mol)	(mL)	(mol)	(%w/w)	(mL)	(mL)	(min)
2.75	0.06	5.5	0.011	0.6	0.033	2.1	0.015	0.6	188	195.4	6
5.5	0.11	11	0.022	1.2	0.067	4.2	0.030	1.3	188	202.8	2
11	0.20	22	0.044	2.4	0.130	8.4	0.060	2.4	188	217.6	0.1
16.5	0.28	33	0.065	3.6	0.200	12.6	0.090	3.4	188	232.4	<0.1

[a] The resulting solution was stirred at room temperature (stirring rate 250–300 rpm). [b] Solvent was removed by centrifugation for 10 min (4000 rpm).

Table S3. Formulations of PUA powders from Desmodur N3300 for preparation in PC/hexane via Suspension Polymerization. [a,b]

[N3300]		N3300		H_2O		Et_3N			PC/hexane	Total Volume	Gelation Time
(% w/w)	(M)	(g)	(mol)	(mL)	(mol)	(mL)	(mol)	(%w/w)	(mL)	(mL)	(min)
5.5	0.11	2.75	0.0055	0.3	0.017	1.05	0.008	1.3	47/141 [c]	191.7	330
11	0.20	5.5	0.011	0.6	0.033	2.1	0.015	2.4	47/141 [d]	195.4	35

[a] The resulting solution was stirred at room temperature (stirring rate 250–300 rpm). [b] Solvent was removed by centrifugation for 10 min (4000 rpm). [c] Volume of hexane was 2.8× the volume of dispersed phase. [d] Volume of hexane was 2.6× the volume of dispersed phase.

Table S4. Formulations of PUA powders from Desmodur N3300 for preparation in PC/hexane via Emulsion Polymerization. [a.]

[N3300]		N3300		H_2O		Et_3N			PC/Hexane	Total Volume	Surfactant (%w/w of Continuous Phase)	Gelation Time
(% w/w)	(M)	(g)	(mol)	(mL)	(mol)	(mL)	(mol)	(%w/w)	(mL/mL)	(mL)		(min)
5.5	0.10	2.75	0.005	0.3	0.02	1.05	0.008	1.3	47 141	191.7	CTAB (0.7)	30
5.5	0.10	2.75	0.005	0.3	0.02	1.05	0.008	1.3	47 141	191.7	CTAB (3.5)	35
11	0.22	5.5	0.011	0.6	0.03	2.1	0.015	2.4	47 141	195.4	CTAB (0.7)	5
11	0.22	5.5	0.011	0.6	0.03	2.1	0.015	2.4	47 141	195.4	CTAB (3.5)	<0.1
5.5	0.10	2.75	0.005	0.3	0.02	1.05	0.008	1.3	47 141	191.7	Nikkol BL-9EX (0.7)	60
5.5	0.10	2.75	0.005	0.3	0.02	1.05	0.008	1.3	47 141	191.7	Nikkol BL-9EX (3.5)	60
11	0.22	5.5	0.011	0.6	0.03	2.1	0.015	2.4	47 141	195.4	Nikkol BL-9EX (0.7)	30
11	0.22	5.5	0.011	0.6	0.03	2.1	0.015	2.4	47 141	195.4	Nikkol BL-9EX (3.5)	15

[a] The resulting mixture was stirred at room temperature (stirring rate 400 rpm).

Table 5. Formulations of PUA powders from Desmodur RE for preparation via Vigorous Agitation or via Precipitation Polymerization. [a,b.]

[TIPM]		TIPM		RE		H_2O		Et_3N			DMF or PC	Hexane	Total Volume	Gelation Time
(% w/w)	(M)	(g)	(mL)	(g)	(mol)	(mL)	(mol)	(mL)	(mol)	(%w/w)	(mL)	(mL)	(mL)	(min)
4	0.10	3.67	0.010	13.59	13.30	0.5	0.028	0.41	0.003	0.3	DMF (81.6)	-	95.81	7
4	0.09	3.67	0.010	13.59	13.30	0.5	0.030	0.3	0.002	0.3	DMF (92.7)	307.2 [c]	414.0	60
4	0.12	2.69	0.007	9.95	9.74	0.4	0.022	0.3	0.002	0.3	PC (47)	141 [d]	198.4	85

[a] The resulting solution was stirred at room temperature (stirring rate 250–300 rpm). [b] Solvent was removed by centrifugation for 10 min (4000 rpm).
[c] Volume of hexane was 3x the volume of dispersed phase. [d] Volume of hexane was 2.5× the volume of dispersed phase.
